# Cannabinoids Inhibited Pancreatic Cancer via P-21 Activated Kinase 1 Mediated Pathway

**DOI:** 10.3390/ijms21218035

**Published:** 2020-10-28

**Authors:** Yang Yang, Nhi Huynh, Chelsea Dumesny, Kai Wang, Hong He, Mehrdad Nikfarjam

**Affiliations:** 1 Department of Surgery, University of Melbourne, Austin Health, Heidelberg, VIC 3084, Australia; yang.yang4@unimelb.edu.au (Y.Y.); Nhi.Huynh@onjcri.org.au (N.H.); watsoncj@unimelb.edu.au (C.D.); kaiw3@student.unimelb.edu.au (K.W.); 2 State Key Laboratory for Biology of Plant Disease and Insect Pests, Institute of Plant Protection, Chinese Academy of Agricultural Sciences, Beijing 100193, China; 3 Tumour Targeting Laboratory, Level 5, ONJCRI, 145 Studley Road, Heidelberg, VIC 3084, Australia

**Keywords:** CBD, THC, PDA, PAK1, PD-L1

## Abstract

The anti-cancer effects of cannabinoids including CBD (Cannabidiol) and THC ((−)-trans-∆9-tetrahydrocannabinol) have been reported in the case of pancreatic cancer (PC). The connection of these cannabinoids to *KRas* oncogenes that mutate in more than 90% of PC, and their effects on PD-L1, a key target of immune checkpoint blockade, have not been thoroughly investigated. Using cell lines and mouse models of PC, the effects of CBD and THC on cancer growth, the interaction between PC cells and a stromal cell, namely pancreatic stellate cells (PSCs), and the mechanism(s) involved were determined by cell-based assays and mouse study in vivo. CBD and THC inhibited the proliferation of PC, PSC, and PSC-stimulated PC cells. They also suppressed pancreatic tumour growth in mice. Furthermore, CBD and/or THC reduced the expression of PD-L1 by either PC or PSC cells. Knockout of p-21 activated kinase 1 (PAK1, activated by *KRas*) in PC and PSC cells and, in mice, dramatically decreased or blocked these inhibitory effects of CBD and/or THC. These results indicated that CBD and THC exerted their inhibitions on PC and PSC via a p-21 activated kinase 1 (PAK1)-dependent pathway, suggesting that CBD and THC suppress *Kras* activated pathway by targeting PAK1. The inhibition by CBD and THC of PD-L1 expression will enhance the immune checkpoint blockade of PC.

## 1. Introduction

Therapeutic resistance and the limited number of effective regimes available for treatment contribute to the poor prognosis of pancreatic cancer (PC) patients. Although the application of immunotherapy, and particularly immune checkpoint blockade, holds promise for improving cancer patient outcomes, single-agent immunotherapy in pancreatic ductal adenocarcinoma (PDA) has not provided significant clinical benefits. This is mainly due to the existence of an immunosuppressive tumour microenvironment (TME) in most human PDA. A highly fibrotic stroma and extensive infiltration of immunosuppressive cells are the major components of this TME [1]. The highly dense stroma provides a barrier to the delivery of cytotoxic agents and limits T cell access to tumour cells [2,3,4]. Pancreatic stellate cells (PSCs) play important roles in the development of the unique desmoplastic reaction in the TME of PDA, by contributing to stromal fibrosis, deregulation of the extracellular matrix and deficient vascularization [5,6]. Thus, modulation of PSCs to reprogram TME and to alter immune suppression should provide great improvements in PDA treatment.

The serine/threonine kinase-p21-activated kinase 1 (PAK1) is one of most important effector proteins of *Kras* which is mutated in over 95% of PDA [7]. Except for the direct effect of PAK1 on cancer growth/metastasis, as identified to other molecules down the *Kras* pathway, we have discovered a role of PAK1 in the tumour immune response and the TME of PDA by showing that the inhibition of PAK1 decreased pancreatic tumour growth and stimulated anti-tumour immunity by down-regulation of PD-L1 (programmed death-ligand 1) expression by PC cells [8,9], that PAK1 is expressed in the PSCs of human PC samples, and that inhibition of PAK1 decreased the proliferation and increased the apoptosis of the PSCs by inhibiting the activation of PSCs [9]. More importantly, we have shown that depletion of PAK1 reduced PSC-stimulated PDA cell proliferation, migration/invasion, suppressed intrinsic and PSC-stimulated PD-L1 expression, and attenuated PSC-mediated protection of PDA cells from cytotoxic lymphocyte-induced cell death, indicating a key role of PAK1 in modulating PSCs activity and anti-tumour immune response in the TME of PC [8].

Plant-derived cannabinoids have been widely investigated for their anti-cancer effects [10]. Cannabidiol (CBD) and (−)-trans-∆9-tetrahydrocannabinol (THC) are the mostly studied cannabinoids from plant extracts. Both THC and CBD inhibited PC cells growth in vitro and in vivo through different mechanisms. THC suppressed PC growth via cannabinoid receptor 2 (CB2) [11] while CBD inhibited PC growth synergistically with gemcitabine through antagonizing the G protein-coupled receptor GPR55 [12]. The mechanism(s) involved in the anti-cancer effects of cannabinoids include direct inhibition on cancer cells and through modulation of tumour immune response [13,14]. Depending on the components and dosages of cannabinoids used and types of receptors activated, cannabinoids execute stimulation or inhibition of anti-tumour immunity [13,14]. However, the role of cannabinoids in regulation of PD-L1 (a key immune checkpoint molecule that negatively regulate T cell activation via a distinct mechanism) expression by cancer cells has not been clearly investigated. Cannabinoids exert anti-proliferation and pro-apoptosis of cancer cells through influencing certain intracellular signalling including ERK- and AKT-dependent pathways [14]. Since PAK1 stimulates cancer cell proliferation, migration/invasion, and survival via ERK- and AKT-dependent pathways [15], it is interesting to determine if cannabinoids act through PAK1 signalling pathway(s).

The aim of this study was to determine the role of cannabinoids (CBD and THC) in proliferation of PC and PSC cells, and in the interaction of PC with PSC cells. The effects of CBD and THC on PD-L1 expression by PC and PSC cells were also investigated. More importantly, the involvement of PAK1 signalling in these effects of CBD and THC was observed.

## 2. Results

### 2.1. Cannabinoids Inhibited Proliferation of PC and PSC Cells and the Interaction of PC with PSC

**Figure 1:**
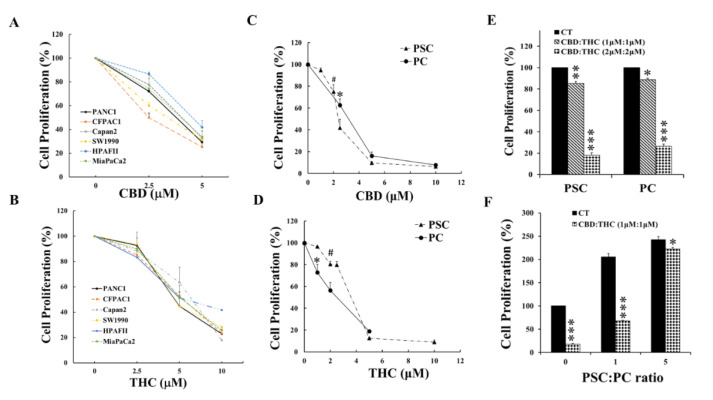
**CBD and THC suppressed proliferation of PC, PSC, and PSC-stimulated PC cells.** A panel of human pancreatic cancer (PC) cells were treated with CBD (**A**) and THC (**B**) for 48 h and cell proliferation was measured by MTT assay as described in the Materials and Methods. Murine PC and pancreatic stellate cells (PSC) were also treated with CBD (**C**) and THC (**D**) for 48 h and cell proliferation was measured by MTT assay. Either PC or PSC cells were treated with CBD and THC at 1:1 ratio as indicated in graph (**E**) for 48 h and cell proliferation was determined by MTT assay. Murine PC (luciferase-labelled) and PSC cells were mixed at the ratios indicated in graph (**F**) and incubated with CBD plus THC at 1:1 ratio, and PC cell proliferation was determined by luciferase activity. The values from non-treated controls were taken as 100%. The data were summarized from three independent sets of experiments. # or *, *p* < 0.05; **, *p* < 0.01; ***, *p* < 0.001 compared to related control. CT: control.

To determine the effects of cannabinoids (CBD and THC) on proliferation of PC and PSC, a panel of human PC cells were incubated with CBD or THC at the concentrations shown in Figure 1A,B and cell proliferation were measured by MTT assay as described in Materials and Methods. Both CBD (Figure 1A) and THC (Figure 1B) suppressed the proliferation of these six human PC cell lines dose-dependently. Compared with THC, CBD showed a more potent inhibitory effect on these human PC cell lines and achieved reduction of proliferation to less than 40% of the control (non-treated cells) at 5 µM while THC at 10 µm. To determine the effects of CBD and THC on PSC cells and to compare the effects of these cannabinoids on PC and PSC cells, murine PC and PCS cells, isolated and characterised as described in previous publications [8,9], were cultured with CBD (Figure 1C) and THC (Figure 1D) for 48 h, followed by MTT assay to measure cell proliferation. CBD and THC inhibited the proliferation of both PC and PSC cells. CBD suppressed the proliferation of PC and PCS cells with similar potency while THC seemed to suppress PC cell proliferation more potently than PSC cells.

One of the cannabis oils used for cancer symptom control in patients, the oil Aurora contains CBD and THC at 1:1 (Cann Group Pty Ltd. Melbourne, VIC, Australia). In consideration of possible future clinical applications of the findings described here, both PC and PSC cells were treated with mixtures of CBD and THC at 1:1 ratio as indicated in Figure 1E,F. The mixtures of CBD and THC (1:1) inhibited the proliferation of both PC and PCS cells (Figure 1E). Furthermore, the mixture of CBD with THC (1 µM:1 µM) suppressed PSC-stimulated PC proliferation (Figure 1F). These results demonstrated the anti-proliferative effects of CBD and THC on PC or PSC cells alone, and on PSC-stimulation of PC cells.

### 2.2. PAK1 Knockout Reduced the Inhibition by Cannabinoids on Proliferation of PC and PSC Cells

**Figure 2:**
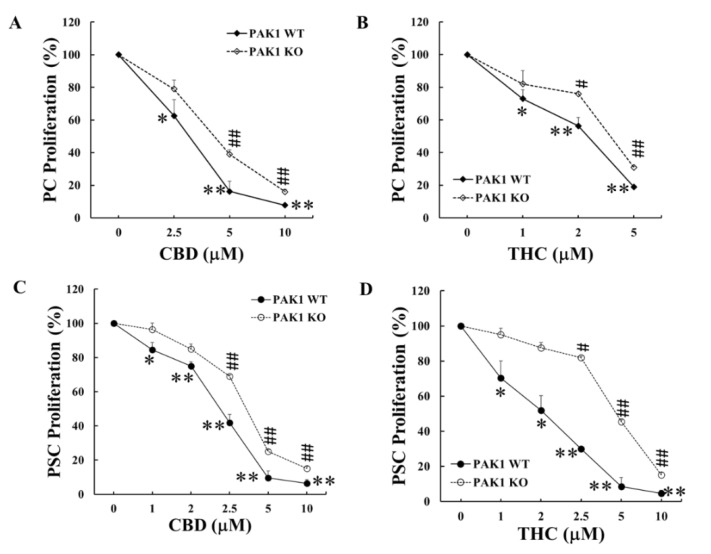
**PAK1 knockout reduced the inhibition of CBD or THC on proliferation of PC and PSC cells.** PAK1 wild type (WT) and knockout (KO) pancreatic cancer (PC) (**A**,**B**) or pancreatic stellate cells (PSC) (**C**,**D**) were treated with CBD (**A**,**C**) or THC (**B**,**D**) for 48 h. Cell proliferation was determined by MTT assay. The values from non-treated controls of either PAK1 WT or KO cells were taken as 100% for the respective cell line. The data were summarized from three independent sets of experiments. *, *p* < 0.05; **, *p* < 0.01 compared to PAK1 WT control. #, *p* < 0.05; ##, *p* < 0.01 compared to PAK1 KO control.

PAK1 stimulates cancer cell proliferation and survival via ERK- and AKT-dependent pathways [15], and both ERK- and AKT-dependent pathways are involved in the anti-proliferation and pro-apoptosis of cancer cells by cannabinoids. PAK1 functions as an important intracellular signal molecule in PC progression. It is critical to investigate the role of PAK1 in the anti-proliferation of PC by cannabinoids. To this end, PAK1 knockout (KO) PC and PSC cells, isolated from murine PC samples as described in previous publication [8], were cultured with CBD and THC to compare the effects of these cannabinoids on proliferation with PAK1 wild type (WT) PC and PSC cells. Both CBD and THC dose-dependently decreased the proliferation of PAK1 WT PC cells (Figure 2A,B). The inhibitory effects of CBD and THC on cell proliferation were reduced in PAK1 KO PC cells (Figure 2A,B) shown by a right shift of the curve. Similarly, CBD and THC suppressed the proliferation of PAK1 WT PSC cells (Figure 2C,D) and the inhibition by CBD or THC was also reduced in PAK1 KO PCS cells (Figure 2C,D), as shown by a right shift of the curve. These results suggested that CBD and THC inhibited proliferation of PC and PCS cells at least partially via a PAK1-dependent pathway.

### 2.3. PAK1 Knockout Blocked the Inhibitory Effect of Cannabinoid Oil on Pancreatic Tumour Growth

**Figure 3:**
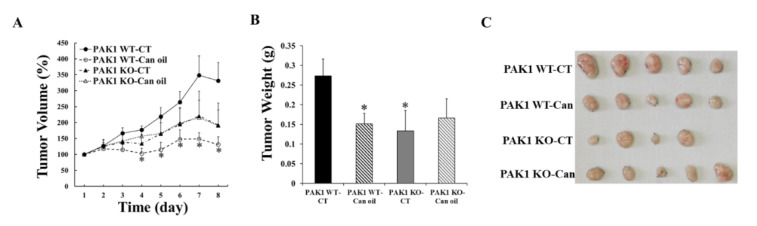
**PAK1 knockout blocked the inhibition of cannabis oil on pancreatic tumour growth.** Murine pancreatic cancer (PC) cells were subcutaneously injected into the flanks of PAK1 wild type (WT, n = 10) and knockout (KO, n = 9) male mice (8-week old). After 5 days of tumour cell injection and when the average tumour volumes reached 50 mm^3^, cannabis oil (Can-oil) containing CBD and THC with 1:1 ratio, was given orally every day for 8 days. Tumour volumes (**A**) were measured daily and tumour weight (**B**) was determined at the end of the experiment. (**C**): photos of tumors dissected at the end of the experiment. CT: control, Can: cannabis oil, Can-oil: cannabis oil. *, *p* < 0.05 compared to non-treated PAK1 WT control.

To investigate the mechanism involved in the inhibitory effect of these cannabinoids in vivo, a syngeneic mouse model of pancreatic cancer was used. Murine PC cells were subcutaneously injected into the flank of C57BL6 mice with either PAK1 WT or PAK1 KO background, and then the mice were treated with cannabis oil by oral gavage daily for eight days. Control mice were given saline orally. In PAK1 WT mice, cannabis oil (CBD:THC at 1:1) reduced the pancreatic tumour growth by decreasing tumour volume (Figure 3A) and tumour weight (Figure 3B). In PAK1 KO mice, the tumour growth was inhibited by PAK1 KO alone without cannabis oil treatment (Figure 3A,B). However, the treatment of cannabis oil could not further reduce the tumour growth in PAK1 KO mice. The result indicated that the inhibition by cannabis oil of pancreatic tumour growth was blocked in PAK1 KO mice, suggesting that these cannabinoids suppressed pancreatic tumour growth in vivo via a PAK1-dependent pathway.

### 2.4. Cannabinoids Decreased the Expressions of PD-L1 by PC and PSC Cells via Down-Regulation of PAK1 Activity

**Figure 4:**
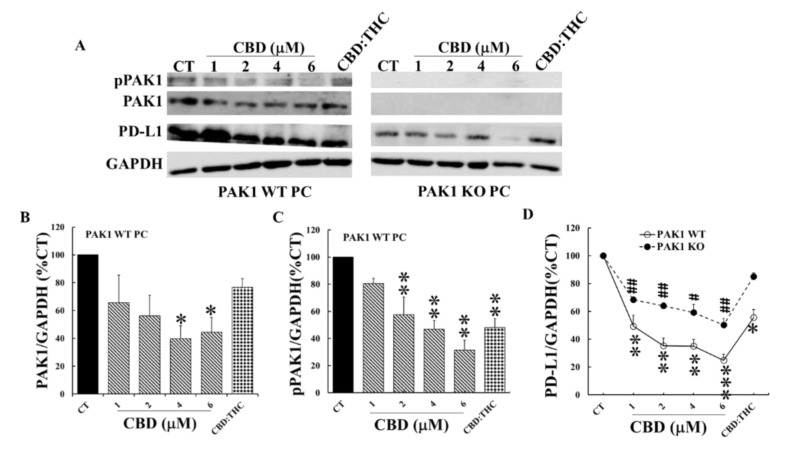
**CBD and THC inhibited PD-L1 expression of PC cells by decreasing PAK1 activity.** PAK1 wild type (WT) and knockout (KO) murine pancreatic cancer (PC) cells were incubated with CBD or CBD plus THC (1 µM:1 µM) for 24 h. The cells were lysed, and the cell lysates were subjected to Western blotting with antibodies against total PAK1, active phosphorylated PAK1 (pPAK1), PD-L1 and GAPDH. Two representative blots were shown in (**A**). The relative expression levels of PAK1 (**B**), pPAK1 (**C**) and PD-L1 (**D**) were calculated from three independent sets of experiments while the values obtained from the non-treated controls of PAK1 WT cells were taken as 100%. In (**D**), the non-treated controls for either PAK1 WT or KO were taken as 100% for the treated WT and KO cell line respectively. CT: control. *, *p* < 0.05; **, *p* < 0.01, ***, *p* < 0.001 compared to the values obtained from the non-treated controls of PAK1 WT cells. #, *p* < 0.05; ##, *p* < 0.01 compared to the values from the non-treated controls of PAK1 KO cells (**D**).

The above results indicated the importance of PAK1 in the inhibition by these cannabinoids of pancreatic cancer growth. Both PAK1 and cannabinoids can exert their effects on cancer by modulation of tumour immune response. We have recently reported that inhibition of PAK1 suppressed pancreatic cancer and stimulated the anti-tumour immunity of PC by down regulation of the expression of PD-L1 (a key target of immune checkpoint inhibition [8]) by cancer cells. Thus, we set-out to determine how cannabinoids affect PD-L1 expression by PC cells and what the role of PAK1 is in this process. To this end, murine PC and PSC cells were incubated with different concentrations of CBD and CBD plus THC at 1:1 ratio (1 µM:1 µM) for 24 h. The levels of PD-L1, as well as total PAK1 and phosphorylated active PAK1 (pPAK1) from these cells, were determined by Western blotting. CBD dose-dependently decreased the protein levels of total PAK1, pPAK1, and PD-L1 in PAK1 WT PC cells (Figure 4). This CBD-induced decrement of PD-L1 was reduced in PAK1 KO PC cells (Figure 4A,D). CBD plus THC treatment did not significantly inhibit PD-L1 in PAK1 KO PC cells (Figure 4). CBD or CBD plus THC inhibited PD-L1 protein expression of PC cells associated with down-regulation of PAK1, suggesting that these cannabinoids down-regulated the expression of PD-L1 by PC cells through a PAK1-mediated pathway.

**Figure 5:**
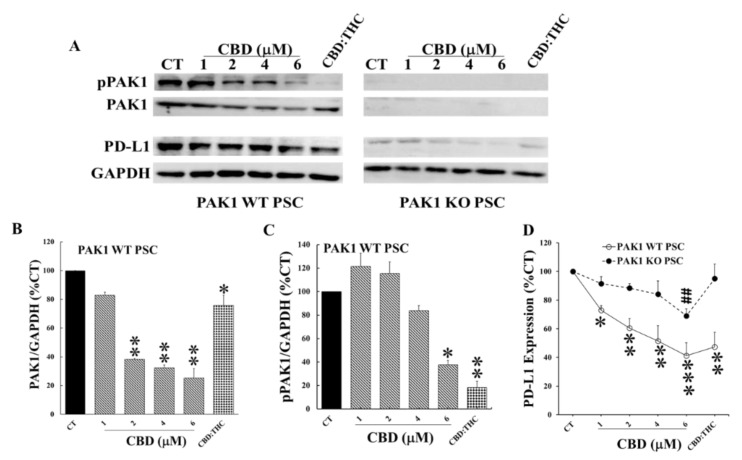
**CBD and THC inhibited PD-L1 expression of PSC cells by decreasing PAK1 activity.** PAK1 wild type (WT) and knockout (KO) murine pancreatic stellate cells (PSC) were incubated with CBD or CBD plus THC (1 µM:1 µM) for 24 h. The cells were lysed, and the cell lysates were subjected to Western blotting with antibodies against total PAK1, active phosphorylated PAK1 (pPAK1), PD-L1 and GAPDH. Two representative blots were shown in (**A**). The relative expression levels of PAK1 (**B**), pPAK1 (**C**) and PD-L1 (**D**) were calculated from three independent sets of experiments while the values obtained from the non-treated controls of PAK1 WT cells were taken as 100%. In (**D**), the non-treated controls for either PAK1 WT or KO were taken as 100% for the treated WT and KO cell line respectively. CT: control. *, *p* < 0.05; **, *p* < 0.01, ***, *p* < 0.001 compared to the values obtained from the non-treated controls of PAK1 WT cells. ##, *p* < 0.01 compared to the values from the non-treated controls of PAK1 KO cells (**D**).

CBD also dose-dependently decreased the protein levels of total PAK1, pPAK1, and PD-L1 in PAK1 WT PSC cells (Figure 5). This CBD-induced decrement of PD-L1 was almost completely blocked in PAK1 KO PSC cells (Figure 5A,D). CBD only significantly inhibited PD-L1 expression of PAK1 KO PSC cells at 6 mM. CBD plus THC treatment did not inhibit PD-L1 expression of PAK1 KO PSC cells (Figure 5). The association of down-regulation of PAK1 with decrement of PD-L1 induced by CBD alone and CBD plus THC suggested that these cannabinoids inhibited the expression of PD-L1 by cancer-associated PSC cells through a PAK1-mediated pathway.

## 3. Discussion

Cannabinoids act through different types of receptors including cannabinoid receptors 1&2 (CB1 and CB2) and another G-protein coupled receptor, GPR55 [13,14]. It is recognised that THC binds to CB1 and CB2 while CBD acts as an antagonist of GPR55, which cause inhibition of ERK- and AKT-dependent pathways to promote apoptosis and to suppress proliferation of cancer cells [16,17]. PAK1 is one of the major effector proteins acting downstream of *KRas* which is mutated in over 95% of PDA. PAK1 stimulates cancer cell proliferation and survival by activation of both ERK- and AKT-dependent pathways [15]. Therefore, it is no coincidence that these cannabinoids (CBD and THC) would act directly or indirectly via a PAK1-mediated pathway to exert their anti-cancer function. Indeed, we have discovered here that both CBD and THC inhibited PC cell proliferation and xenografted tumour growth through a PAK1-mediated pathway as depletion of PAK1 by knockout reduced (Figure 2) or blocked (Figure 3) the inhibitory effects of these cannabinoids on PC. These results confirmed that CBD and/or THC suppressed PC growth at least partially through inhibition of PAK1. The results from the flank tumor mouse study here will be further validated in our orthotopic pancreatic cancer models to investigate the mechanism(s) of these cannabinoids more thoroughly to gain more conclusive knowledge.

The activation of GPR55 receptor leads to stimulation of RhoA, CDC42, and Rac1 [18], which belong to the Ras-super family of small G-proteins and activate PAK1. CBD, acting as an antagonist to GPR55 receptor, can decrease the stimulation of RhoA, CDC42 and Rac1by GPR55, and thus reduce the activation of PAK1. Recently a CB2-GPR55 heteromer has been identified in cancer cells and these CB2-GPR55 heteromers drive biphasic signalling responses as opposed to the individual receptors via cross-antagonism [19]. Therefore, THC can also exert an antagonist-like effect on GPR55 via binding to the CB2 receptor, leading to the downregulation of PAK1 induced by GPR55-stimulation of RhoA, CDC42, and Rac1.

Cannabinoids appear in part to exhibit their anti-cancer effects through modulation of tumour immune responses. Both CBD and THC have been reported to cause changes in T helper cell profiles leading to suppression of cytotoxic T cell function [13,16]. The anti-inflammation effects of cannabinoids contribute to the immunosuppressive tumour microenvironment on one hand, but can also prevent carcinogenesis by suppressing chronic inflammation [20,21] on the other hand. The immunosuppressive effects of cannabinoids are mainly mediated by CB2 receptors which are extensively expressed in immune cells [13]. However, cannabinoids can also elicit anti-tumour immune responses via different mechanisms. THC and CBD have been reported to stimulate the expression of the intercellular adhesion molecule 1 (ICAM-1) on lung cancer cells to enhance the susceptibility of cancer cells towards lysis by lymphokine-activated killer cells [22]. In experimental skin cancer, THC decreased infiltration of macrophages and neutrophils, which was associated with tumour regression [23]. More recently, CBD has been shown to reduce the PD-L1 expression of glioblastoma cells, which was associated with the increased apoptosis of these cells [24]. PD-L1 overexpressed by cancer cells, binds to programmed cell death protein-1 (PD-1) on T cells and causes apoptosis and inactivation of T cells. The antibody-mediated blockade of these immune checkpoint molecules leads to T cell activation and enhanced anti-tumour immunity [25,26]. Although anti-PD-1/PD-L1 immunotherapy has shown long-lasting efficacy in many types of human cancers [27], the response to immune checkpoint blockade is poor in PC [28]. We have shown here that CBD and THC reduced the expression of PD-L1 by both PC and PSC cells through down-regulation of PAK1 (Figure 4 and Figure 5). The inhibition of PD-L1 expression by CBD and THC will enhance the anti-tumour immune response induced by immune checkpoint blockade in pancreatic cancer.

More importantly we have demonstrated the inhibitory effects of CBD and THC on PSC cells, which were more dependent on PAK1 as the inhibition by CBD and/or THC of proliferation and PD-L1 expression of PSC cells were almost completely blocked in PAK1 knockout PSC cells (Figure 2 and Figure 5). PSC cells play a key role in the desmoplastic reaction in the TME of PDA [29,30,31], which contributes significantly to the aggressiveness and therapy resistance of PDA [30,32]. The modulation of PSC cells will contribute to reprogramming the TME of PC to reduce immunosuppression. We have recently reported that PAK1 plays a key role in modulating PSC activity to promote anti-tumour immunity in PDA [8]. CBD and THC, by acting through PAK1-mediated pathways, inhibited not only PSC proliferation and expression of PD-L1, but also PSC activity towards stimulation of PC. These effects of CBD and/or THC will contribute to modulation of the TME of PC to enhance the anti-tumour immunity.

In conclusion, we have reported for the first time that CBD and/or THC not only inhibited PC cells or PSC cells separately, but also suppressed the PSC activity towards stimulation of PC. CBD and THC exerted their inhibitory effects on PC via a PAK1-dependent pathway, suggesting that CBD and THC suppress *Kras* activated pathway by targeting PAK1. The suppression of PD-L1 expression by these cannabinoids could enhance the immune checkpoint blockade in PC.

## 4. Materials and Methods

### 4.1. Cell and Reagents

Human pancreatic cancer cells, PANC1, CFPAC1, Capan2, SW1990, HPAF11, and MiaPaCa2 were purchased from ATCC (American Type Culture Collection, In vitro technologies, Melbourne, VIC, Australia). Murine tumour-associated pancreatic stellate cells (PSC) were isolated from tumour fragments of KPC (*LSL-Kras^G12D/+^; LSL-Trp53^R172H/+^; Pdx-1-Cre*) mice by the outgrowth method [9]. The luciferase-labelled murine pancreatic cancer (PC) cell line, TB33117 was given by Dr David Herrmann and Dr Paul Timpson (Invasion, Metastasis Group, Cancer Research Program, The Kinghorn Cancer Centre, Garvan Institute of Medical Research, Darlinghurst, Sydney, NSW, Australia). Purified CBD and THC, and cannabis oil containing CBD (13 mg/mL) and THC (13mg/mL), were provided by Cann Group Limited (Mount Waverly, Melbourne, VIC, Australia). Cells were cultured in Dulbecco’s Modified Eagle’s Medium (DMEM) supplemented with 10% FBS (fetal bovine serum: Hyclone Laboratories Inc., Melbourne, VIC, Australia) in a 37 °C incubator with a humidified atmosphere of 5% CO_2_.

### 4.2. Cell Proliferation Assay

Human and murine PC cells, and murine PSC cells (2−5 × 10^3^ cells/well) were incubated in 5% FBS DMEM with different concentrations of CBD or THC, and CBD plus THC at 1:1 ratio for 48 h in 96-well plates. By the end of the culture, cell proliferation was measured by MTT assay. Luciferase-labelled murine PC cell, TB33117 (2 × 10^3^ cells/well) were mixed with different ratios of murine PSC cells as indicated in the text, and cultured with CBD (1 µM) plus THC (1 µM) for 48 h. The PC cell proliferation was determined by the luciferase activity as assessed by the Dual-Luciferase^®^ Reporter Assay (Promega, Sydney, NSW, Australia) following the manufacturer’s instructions. Luciferase intensity was measured with a FLUOstar OPTIMA microplate reader (BMG Labtech, Melbourne, VIC, Australia).

### 4.3. Mouse Study

All mouse experiments were approved by the Austin Health Animal Ethics Committee (approval ID: A2018/05509; approved on May 4, 2018). Experimental mice were housed in the BioResource Facility at Austin Health and monitored according to health criteria. Murine PC cells TB33117 (5 × 10^3^ cells/100 µL culture medium) were subcutaneously injected into flanks of 6-week old, male C57BL6 of PAK1 wild type (WT) and knockout (KO) mice. When a tumour size reached 50 mm^3^ (in about 5 days), cannabis oil (250 µL, CBD to THC at 1:1) was given by oral gavage daily for 8 days. The control mice were given same volume of saline. Tumour growth was determined by tumour volume measured by a calliper daily and by tumour weight assayed at the end of the experiment.

### 4.4. Western Blot

PC and PSC cells were incubated with CBD or CBD plus THC at 1:1 ratio for 24 h. At the end of the incubation, cells were lysed in SDS sample buffer and the resultant cell lysates were electrophoresed on 10% SDS-PAGE followed by Western blotting with antibodies against PD-L1, phosphorylated PAK1 (pPAK1), total PAK1, and GAPDH (Cell Signalling Technology, Arundel, QLD, Australia). The relative amount of each protein was calculated as the ratio of the density of its band to the density of the GAPDH band.

### 4.5. Statistical Analysis

All values are expressed as mean ± standard error. The in vitro data are summarized from three independent experiments. The in vivo data were collated according to numbers of tumour samples. Data were analyzed by one-way ANOVA or student’s *t*-test (SPSS, IBM, New York, NY, USA). Differences between two means with *p* < 0.05 were considered significant.

## Abbreviations

CBD	cannabidiol
THC	(−)-trans-∆9-tetrahydrocannabinol
PDA	pancreatic ductal adenocarcinoma
PC	pancreatic cancer
PSC	pancreatic stellate cell
PAK1	p-21 activated kinase 1
TME	tumour microenvironment
PD-L1	programmed death-ligand

## References

[1] Laklai H., Miroshnikova Y.A., Pickup M.W., Collisson E.A., Kim G.E., Barrett A.S., Hill R.C., Lakins J.N., Schlaepfer D.D., Mouw J.K. (2016). Genotype tunes pancreatic ductal adenocarcinoma tissue tension to induce matricellular fibrosis and tumor progression. Nat. Med..

[2] Feig C., Jones J.O., Kraman M., Wells R.J., Deonarine A., Chan D.S., Connell C.M., Roberts E.W., Zhao Q., Caballero O.L. (2013). Targeting CXCL12 from FAP-expressing carcinoma-associated fibroblasts synergizes with anti-PD-L1 immunotherapy in pancreatic cancer. Proc. Natl. Acad. Sci. USA.

[3] Ozdemir B.C., Pentcheva-Hoang T., Carstens J.L., Zheng X., Wu C.C., Simpson T.R., Laklai H., Sugimoto H., Kahlert C., Novitskiy S.V. (2014). Depletion of carcinoma-associated fibroblasts and fibrosis induces immunosuppression and accelerates pancreas cancer with reduced survival. Cancer Cell.

[4] Provenzano P.P., Cuevas C., Chang A.E., Goel V.K., Von Hoff D.D., Hingorani S.R. (2012). Enzymatic targeting of the stroma ablates physical barriers to treatment of pancreatic ductal adenocarcinoma. Cancer Cell.

[5] Vonlaufen A., Joshi S., Qu C., Phillips P.A., Xu Z., Parker N.R., Toi C.S., Pirola R.C., Wilson J.S., Goldstein D. (2008). Pancreatic stellate cells: Partners in crime with pancreatic cancer cells. Cancer Res..

[6] Vennin C., Murphy K.J., Morton J.P., Cox T.R., Pajic M., Timpson P. (2018). Reshaping the Tumor Stroma for Treatment of Pancreatic Cancer. Gastroenterology.

[7] Biankin A.V., Waddell N., Kassahn K.S., Gingras M.C., Muthuswamy L.B., Johns A.L., Miller D.K., Wilson P.J., Patch A.M., Wu J. (2012). Pancreatic cancer genomes reveal aberrations in axon guidance pathway genes. Nature.

[8] Wang K., Zhan Y., Huynh N., Dumesny C., Wang X., Asadi K., Herrmann D., Timpson P., Yang Y., Walsh K. (2020). Inhibition of PAK1 suppresses pancreatic cancer by stimulation of anti-tumour immunity through down-regulation of PD-L1. Cancer Lett.

[9] Yeo D., Phillips P., Baldwin G.S., He H., Nikfarjam M. (2017). Inhibition of group 1 p21-activated kinases suppresses pancreatic stellate cell activation and increases survival of mice with pancreatic cancer. Int. J. Cancer.

[10] Javid F.A., Phillips R.M., Afshinjavid S., Verde R., Ligresti A. (2016). Cannabinoid pharmacology in cancer research: A new hope for cancer patients?. Eur. J. Pharmacol..

[11] Carracedo A., Gironella M., Lorente M., Garcia S., Guzman M., Velasco G., Iovanna J.L. (2006). Cannabinoids induce apoptosis of pancreatic tumor cells via endoplasmic reticulum stress-related genes. Cancer Res..

[12] Ferro R., Adamska A., Lattanzio R., Mavrommati I., Edling C.E., Arifin S.A., Fyffe C.A., Sala G., Sacchetto L., Chiorino G. (2018). GPR55 signalling promotes proliferation of pancreatic cancer cells and tumour growth in mice, and its inhibition increases effects of gemcitabine. Oncogene.

[13] Vecera L., Gabrhelik T., Prasil P., Stourac P. (2020). The role of cannabinoids in the treatment of cancer. Bratisl. Lek. Listy.

[14] Hinz B., Ramer R. (2019). Anti-tumour actions of cannabinoids. Br. J. Pharmacol..

[15] Huynh N., Liu K.H., Baldwin G.S., He H. (2010). P21-activated kinase 1 stimulates colon cancer cell growth and migration/invasion via ERK- and AKT-dependent pathways. Biochim. Biophys. Acta.

[16] Sledzinski P., Zeyland J., Slomski R., Nowak A. (2018). The current state and future perspectives of cannabinoids in cancer biology. Cancer Med..

[17] Pellati F., Borgonetti V., Brighenti V., Biagi M., Benvenuti S., Corsi L. (2018). *Cannabis sativa* L. and Nonpsychoactive Cannabinoids: Their Chemistry and Role against Oxidative Stress, Inflammation, and Cancer. Biomed. Res. Int..

[18] Lauckner J.E., Jensen J.B., Chen H.Y., Lu H.C., Hille B., Mackie K. (2008). GPR55 is a cannabinoid receptor that increases intracellular calcium and inhibits M current. Proc. Natl. Acad. Sci. USA.

[19] Moreno E., Andradas C., Medrano M., Caffarel M.M., Perez-Gomez E., Blasco-Benito S., Gomez-Canas M., Pazos M.R., Irving A.J., Lluis C. (2014). Targeting CB2-GPR55 receptor heteromers modulates cancer cell signaling. J. Biol. Chem..

[20] Chan P.C., Sills R.C., Braun A.G., Haseman J.K., Bucher J.R. (1996). Toxicity and carcinogenicity of delta 9-tetrahydrocannabinol in Fischer rats and B6C3F1 mice. Fundam. Appl. Toxicol..

[21] Liu W.M., Fowler D.W., Dalgleish A.G. (2010). Cannabis-derived substances in cancer therapy--an emerging anti-inflammatory role for the cannabinoids. Curr. Clin. Pharmacol..

[22] Haustein M., Ramer R., Linnebacher M., Manda K., Hinz B. (2014). Cannabinoids increase lung cancer cell lysis by lymphokine-activated killer cells via upregulation of ICAM-1. Biochem. Pharmacol..

[23] Glodde N., Jakobs M., Bald T., Tuting T., Gaffal E. (2015). Differential role of cannabinoids in the pathogenesis of skin cancer. Life Sci..

[24] Ivanov V.N., Wu J., Wang T.J.C., Hei T.K. (2019). Inhibition of ATM kinase upregulates levels of cell death induced by cannabidiol and gamma-irradiation in human glioblastoma cells. Oncotarget.

[25] Topalian S.L., Hodi F.S., Brahmer J.R., Gettinger S.N., Smith D.C., McDermott D.F., Powderly J.D., Carvajal R.D., Sosman J.A., Atkins M.B. (2012). Safety, activity, and immune correlates of anti-PD-1 antibody in cancer. N. Engl. J. Med..

[26] Brahmer J.R., Tykodi S.S., Chow L.Q., Hwu W.J., Topalian S.L., Hwu P., Drake C.G., Camacho L.H., Kauh J., Odunsi K. (2012). Safety and activity of anti-PD-L1 antibody in patients with advanced cancer. N. Engl. J. Med..

[27] Emens L.A., Ascierto P.A., Darcy P.K., Demaria S., Eggermont A.M.M., Redmond W.L., Seliger B., Marincola F.M. (2017). Cancer immunotherapy: Opportunities and challenges in the rapidly evolving clinical landscape. Eur. J. Cancer.

[28] Myint Z.W., Goel G. (2017). Role of modern immunotherapy in gastrointestinal malignancies: A review of current clinical progress. J. Hematol. Oncol..

[29] Apte M.V., Wilson J.S. (2003). Stellate cell activation in alcoholic pancreatitis. Pancreas.

[30] Apte M.V., Park S., Phillips P.A., Santucci N., Goldstein D., Kumar R.K., Ramm G.A., Buchler M., Friess H., McCarroll J.A. (2004). Desmoplastic reaction in pancreatic cancer: Role of pancreatic stellate cells. Pancreas.

[31] Haber P.S., Keogh G.W., Apte M.V., Moran C.S., Stewart N.L., Crawford D.H., Pirola R.C., McCaughan G.W., Ramm G.A., Wilson J.S. (1999). Activation of pancreatic stellate cells in human and experimental pancreatic fibrosis. Am. J. Pathol..

[32] Olive K.P., Jacobetz M.A., Davidson C.J., Gopinathan A., McIntyre D., Honess D., Madhu B., Goldgraben M.A., Caldwell M.E., Allard D. (2009). Inhibition of Hedgehog signaling enhances delivery of chemotherapy in a mouse model of pancreatic cancer. Science.

